# Social Relationships Are Key to Health, and to Health Policy

**DOI:** 10.1371/journal.pmed.1000334

**Published:** 2010-08-31

**Authors:** The PLoS Medicine Editors

## Abstract

The *PLoS Medicine* editors argue for the need to fundamentally rethink how societies can look beyond the “medical” causes of disease in an effort to promote health and well-being.

Evidence from observational studies has documented the association between social relationships and beneficial effects on health outcomes, such as mortality [1]. However, the precise size of this effect, and of which aspects that form part of social relationships are most strongly linked with positive outcomes, remain unclear. Now, a systematic review and meta-analysis of the literature [2] sheds further light on these questions. The systematic review, published in *PLoS Medicine* in July 2010, retrieved data from a large body of literature—148 studies involving 308,849 participants. The researchers examined studies carried out in both community populations and patient samples, and examined only the “hardest” endpoint—mortality (excluding studies in which only suicide or injury-related mortality was reported). The researchers reported that stronger social relationships were associated with a 50% increased chance of survival over the course of the studies, on average. The effect was similar for both “functional” (e.g., the receipt or perception of receipt of support within a social relationship) and “structural” measures of relationships (e.g., being married, living alone, size of social networks).

Quite remarkably, the degree of mortality risk associated with lack of social relationships is similar to that which exists for more widely publicized risk factors, such as smoking. Arguably, such a level of risk deserves attention at the highest possible level in determination of health policy. In the UK, the Strategic Review of Health Inequalities (Marmot Review) [3] does indeed emphasize the need to reduce social isolation as a crucial means toward addressing health disparities, particularly in the most deprived groups. As mechanisms toward this goal, the Report recommends providing support for community groups with long-term funding, and stresses the importance of addressing social cohesion by ensuring integrated transport links and street safety. However, such social interventions have traditionally not been seen as falling under the rubric of health policy. It is illuminating that of the 22 itemized components of ill-health–prevention expenditure for England covering 2006/2007, and listed in the Marmot Review [3], interventions addressing social integration or isolation do not appear at all (the top five include maternal and child health, family planning and counseling; health protection agency funding; immunization; obesity/diet/lifestyle funding; and smoking cessation services). It remains to be seen whether future governmental ill-health–prevention strategies will reflect the goals and priorities of this Report and include funding for the types of social interventions suggested above.

However, even if community interventions for promoting cohesive social relationships were to be prioritized as a part of national social and health policies, governments around the world are currently facing the challenge of drastic cuts to public spending in order to reduce deficits. In the UK [4], government expenditure will be cut by an additional UK£40bn per year, over and above savings set out by the previous government. Although it has been declared that spending on health care will be protected, social care and other public services will not, and these as well as other government departments are tasked with achieving 25% reductions in their expenditure over the next four years. Such an approach to addressing an economic recession risks taking a short-sighted view, as argued in a recent economic analysis [5]. David Stuckler and colleagues examined, at a macroeconomic level, the associations between social care spending and health outcomes. Their assessment clearly outlines the inverse correlation between increased welfare spending, at a country-by-country level, and national mortality rates, an association explained in only small part by the confounding effect of overall national wealth. As Stuckler and colleagues acknowledged, the recent and urgent need to restrict spending ignores the complex interconnectivities between social care and health. Instead, they urge novel approaches, seeing the current crisis as “….an opportunity to reorganise provision of services to those in need, creating a broader set of services that reflect the increasingly complex needs of a society…”

But what might effective services oriented around the core goals of reducing social isolation, and encouraging supportive relationships in society, look like? It is daunting to acknowledge that the underlying mechanisms are unclear through which social relationships affect health, let alone know how to design effective social interventions at a population level that will result in improved health outcomes. It is not possible to randomize individuals to have friends, nor to establish and maintain close and supportive relationships with a partner, although numerous research studies have been done to test the benefits associated with externally provided social support. Although the findings of some trials are encouraging (e.g., a study providing peer support to women identified as at high risk for postnatal depression [6]), some studies (e.g., aimed at promoting social support as part of treatment after heart attack or stroke [7],[8], and befriending interventions in dementia [9]) have failed to find beneficial effects on outcomes such as survival or caregiver depression. Such research-led interventions are commonly intensive and expensive, and do not closely mimic the experiences and interactions that individuals have as part of their naturally occurring social relationships. Some insights into the approaches that may work at a community level come from an evaluation of the UK scheme “Partnership for Older People Projects,” within which £60m was made available at a local level for services, mainly addressing social isolation among elderly people [10]. Funded projects encompassed a broad range of initiatives, such as befriending schemes, leisure and library schemes, social and caregiver support for individuals just discharged from the hospital, and intensive home support for people thought to be at serious risk of hospital admission. The researchers calculated that, for every £1 spent on the trial projects, local hospitals would have saved around £1.20 in emergency hospital care, and that overnight stays in hospital were reduced by approximately 47%.

Findings such as these, and of the systematic review published in *PLoS Medicine* [2], argue strongly for the need to fundamentally rethink how societies can look beyond the “medical” causes of disease in an effort to promote health and well-being, and that governments can, and should, do much toward this goal—even during a period of economic crisis.
